# High Temperature-Resistant Transparent Conductive Films for Photoelectrochemical Devices Based on W/Ag Composite Nanonetworks

**DOI:** 10.3390/nano13040708

**Published:** 2023-02-12

**Authors:** Menghan Liu, Peiling Ren, Hu Qiao, Miaomiao Zhang, Wenxuan Wu, Baoping Li, Hongjun Wang, Daobin Luo, Jianke Liu, Youqing Wang

**Affiliations:** 1 Research Center for Semiconductor Materials and Devices, Shanxi University of Science and Technology, Xi’an 710021, China; 2 School of Mechatronic Engineering, Xi’an Technological University, Xi’an 710021, China

**Keywords:** Ag nanowires, W nanowires, transparent conductive films, thermal stability, semi-coherent interface, electrospinning

## Abstract

The traditional Ag nanowire preparation means that it cannot meet the demanding requirements of photoelectrochemical devices due to the undesirable conductivity, difficulty in compounding, and poor heat resistance. Here, we prepared an Ag nanonetwork with superior properties using a special template method based on electrospinning technology. The transparent conductive films based on Ag nanonetworks have good transmittance in a wide range from ultraviolet to visible. It is important that the films have high operability and are easy to be compounded with other materials. After compounding with high-melting-point W metal, the heat-resistance temperature of the W/Ag composite transparent conductive films is increased by 100 °C to 460 °C, and the light transmission and electrical conductivity of the films are not significantly affected. All experimental phenomena in the study are analyzed theoretically. This research can provide an important idea for the metal nanowire electrode, which is difficult to be applied to the photoelectrochemical devices.

## 1. Introduction

Transparent conductive films (TCFs) are an important part of many optoelectronic devices and can be used as electrodes, optical windows or screens [1,2]. In different application fields, in addition to excellent light transmittance and electrical conductivity, the device also requires TCF to have good thermal stability, mechanical stability, flexibility, and light transmittance for specific wavelength bands. Depending on the materials used, TCFs can be divided into three categories: (1) TCFs based on transparent conductive oxides (TCOs), such as FTO (fluorine-doped tin oxide)/ITO (indium tin oxide). FTO/ITO is currently the most widely used TCF, with excellent stability, and it is suitable for large-scale production. FTO/ITO has more than 80% transmittance in the visible region, but cannot transmit ultraviolet (UV) light, which makes it impossible to apply to UV devices. (2) TCFs based on carbon materials, such as graphene or carbon nanotubes. This type of TCF has excellent thermal stability; however, achieving a high level of light transmittance and electrical conductivity at the same time is difficult, and carbon materials also absorb UV light. (3) TCF based on metal nanowires (NWs). The metal material itself has extremely low resistivity, and using a reasonable design, we can obtain the TCF with the best conductivity. In addition, TCFs based on metal NWs have unique advantages in broadband transmission. Among the many metal materials used to prepare TCF (such as Ag, Cu, Au and Al), Ag NWs (AgNWs) are the representative materials with the largest development potential [3,4].

Efficient chemical liquid–phase synthesis is the traditional method for preparing AgNWs, but the AgNWs obtained using this method are prone to introducing impurities, the aspect ratio of the NWs is insufficient, and the intersection impedance is very large. Researchers have tried different methods to prepare and optimize the AgNW-based TCFs (AgTCFs). Jinhwan Lee et al. [5] prepared ultra-long AgNWs using a simple multistep growth method and mask plate, and discussed the connection of NWs at the intersection. Chen et al. [6] prepared Ag/Ni metal-mesh with low surface roughness based on hybrid printing of Ag nanoparticle ink and electroplating of Ni. In order to eliminate the adverse effects on the conductivity and carrier transport of the films, the researchers proposed to use a rapid electrochemical cleaning strategy to remove PVP (Polyvinyl Pyrrolidone) ligands from the AgNW surface [7]. Jiang et al. [8] prepard Cu/Ag core/shell electrospun nanofibers as highly stable and flexible transparent conductive electrodes for optoelectronic devices. In addition, obtaining pure AgNW using physical methods has been extensively studied.

On the other hand, AgTCF has a key weakness: it cannot withstand high temperatures. After the material is nanosized, the melting point will drop sharply, and the heat-resistant temperature of conventional AgNWs can even be as low as 300 °C, which severely limits its application in high-temperature processing devices. In many devices, such as dye-sensitized solar cells (DSSCs), the electrodes are required to withstand at least 450 °C. In recent years, nanocomposites and two-phase interfaces have attracted much attention due to the grain growth and overall stability of nanocrystals. Interfacial control has gradually become an important way to improve the heat resistance of two-phase bimetallic nanocomposites [9,10]. Zheng et al. [11] used the superposition rolling method to prepare Cu-Nb nanomultilayer composite, and found that the formation of low-energy stable semi-coherent interface between Cu-Nb makes it have excellent thermal stability. In the comparative study of Pb/Al nanoparticles samples by Sheng [12], it is found that Pb particles have obvious overheating phenomenon when the Pb/Al interface is semi-coherent. Zhang et al. [13] successfully observed the phenomenon of overheating in Pb/Al thin films, which is because the epitaxial interface constraint formed by Al/Pb/Al inhibits the growth of liquid phase nuclei during the melting process, which indicates once again that the low energy interface plays an important role in the overheating behavior. Therefore, optimizing the transparency and conductivity of AgTCF while increasing its heat-resistance temperature is critical for its application in a wide range of devices.

In this work, a novel physical template method based on electrospinning and magnetron sputtering was employed to prepare Ag and W composite NW networks. Using quartz as substrate, the obtained AgTCF exhibited good transmittance in a very wide range, from UV to the visible region. This makes it possible to apply it in both visible and UV devices. Thanks to the good connection at the crossover of AgNWs, the sheet resistance of the film can be as low as 4.2 Ω/sq. In addition, the presence of the Ag/W interface increases the heat resistance of the film to 460 °C. This depends on three main points: (1) the semi-coherent lattice relationship at the interface, (2) the thermal expansion, and (3) the chemical bonding at the interface. The improved thermal resistance offers the possibility of AgTCF in high temperature devices. We believe that the above research ideas are important references for the application of AgNW electrodes in devices such as DSSCs and photoelectrochemical UV detectors.

## 2. Experimental Part

All chemicals are of analytical grade.

### 2.1. Preparation of W and Ag Composite Nanonetwork TCFs

**Figure 1:**
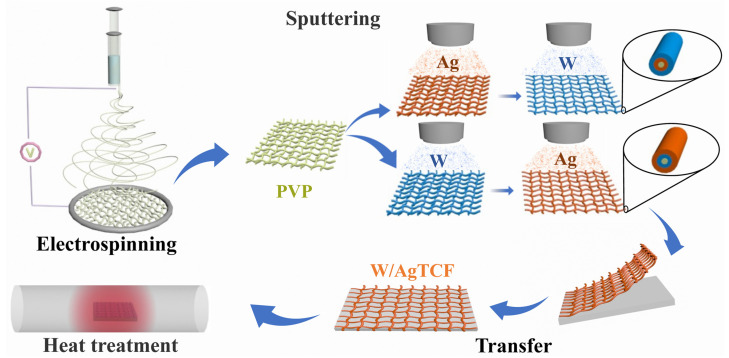
Schematic diagram of the experimental flow for the preparation of W/Ag (or Ag/W) composite nanonetwork TCFs.

The whole preparation process of TCF is shown in Figure 1. The entire preparation process of quartz-based reticulated metal NW consists of electrospinning, magnetron sputtering and film transfer. Electrospinning is a method to obtain nanofibers by spraying and stretching polymer solution under the action of gravity and electric force in an electrostatic field. The experimental device consists of a high-voltage power supply, a glass syringe, a needle and a circular metal ring. In this experiment, PVP polymer templates were prepared by electrospinning to be used as templates for subsequent sputtering of metal NWs.

First, PVP polymer solution was prepared. The 5.4 mL anhydrous ethanol was poured into a clean beaker, and 0.35 g PVP powder was slowly added into anhydrous ethanol, and stirred for 30 min. Then, pour the prepared PVP solution into the syringe, and place the needle downward and connect it with a high-voltage power supply, so that the solution drops naturally under the action of gravity. The nano-fibers ejected will be overlapped on the circular metal ring below the nozzle of the needle. Up to this point, the PVP NW network template was obtained. The density of the template can be controlled by the spinning time.

Magnetron sputtering is used to deposit metal layers. The rings with PVP nano-networks obtained in the previous step were placed under the metal target as is. After the sputtering process, metal atoms were deposited on the surface of the PVP template, resulting in a skeletonized film composed of a network of metal NWs with the same morphology as the PVP template. In this work, we choose Ag and W metals to deposit thin films on the PVP template, either by sputtering a single component or by sputtering multiple times to form a composite NW network. The obtained W NWs were abbreviated as WNWs. The samples obtained by sputtering Ag first and then W were referred to as Ag/WNWs (and accordingly, Ag/WTCFs). The samples obtained by sputtering W first and then Ag are referred to as W/AgNWs (and accordingly, W/AgTCFs).

The sputtering parameters were: DC sputtering mode, sputtering power 150 W, working pressure 0.5 Pa. Sputtering time was adjusted according to experimental requirements.

### 2.2. Transfer Metal Nanonetworks onto Quartz Substrate

The metal nanonetworks obtained using this method are naturally attached to the sample collection ring and can be manipulated very conveniently with tweezers. For transfer, it is sufficient to cover the ring with the metal nanonetwork attached on a substrate smaller than the ring and then remove the excess. The quartz glass is ultrasonically cleaned with acetone, ethanol and deionized water in turn before attaching the metal NW. Since PVP polymer is soluble in alcohol, an appropriate amount of alcohol is dropped on the quartz glass before sticking, and the metal nanonetwork is adhered to the wet quartz glass. After drying at room temperature for 1 h, subsequent operations or measurements can be performed.

### 2.3. Characterization

The transmittance of TCFs was measured using a dual-beam UV–vis photometer. Four probes were used to measure the sheet resistance of the samples. In order to reduce the error, several groups of results were selected to calculate the average value. Field emission scanning electron microscope (FESEM, Hitachi S-4800) and an optical microscope were used to characterize the morphology and structure of the samples. To study the heat resistance of the films, the samples were annealed in a KSL-1100X muffle furnace at different temperatures. We did not remove the PVP before all heat-resistance testing.

For electrospinning technology, the preparation parameters must be precisely controlled to obtain well reproducible samples. In this study, we obtained several samples with different NW densities by varying the spinning time (other conditions being the same) and investigated their heat resistance to obtain representative conclusions and to verify the factors affecting the heat-resistance temperature.

## 3. Results and Discussion

### 3.1. The Ag Nanonetwork TCF Prepared Using Template Method

**Figure 2:**
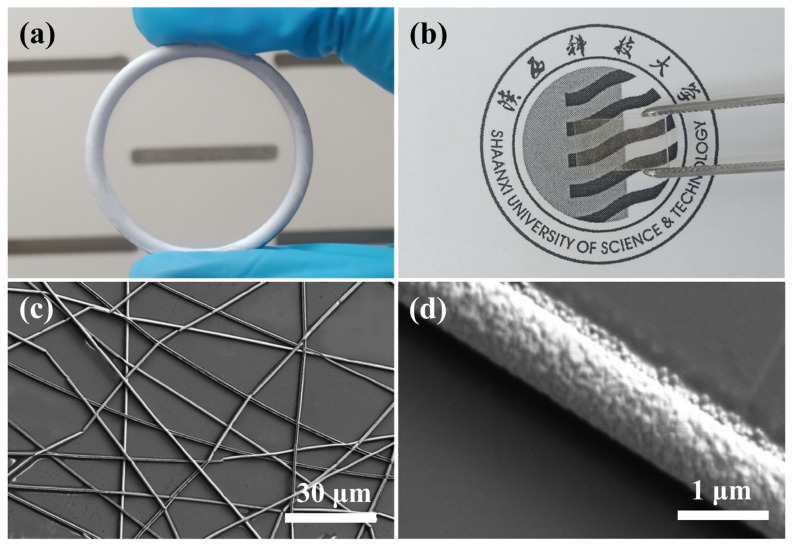
(**a**) Thin Ag film attached to the collection ring. (**b**) The AgTCF obtained by transferring AgNWs to a quartz substrate. (**c**) FESEM image of Ag nanonetworks. (**d**) FESEM morphology of single AgNW.

Figure 2a shows the obtained network of AgNWs attached to the metal collecting ring. It can be seen that there is a very homogeneous and almost invisible film inside the ring, which can reach a size of more than 8 cm. The metal films attached to the rings can be easily transferred to any substrate, which is the advantage of this particular method. Figure 2b shows a photo of the AgNW network after it has been attached to a quartz substrate. In addition, this strategy is applicable to the preparation of flexible TCF, which only requires the selection of a flexible substrate. A small amount of alcohol dripped on the substrate during the transfer process can effectively increase the adhesion between the film and the substrate. The PVP template does not significantly affect the light transmission and electrical conductivity of the film and can be removed on a case-by-case basis. As shown in Figure 2c,d, we have characterized the microscopic morphology of AgNWs using SEM. The AgNWs tightly attached to the substrate surface have three significant advantages: (1) the length of AgNWs can reach the centimeter scale (due to experimental constraints, we can only measure the approximate scale), which is much larger than the samples prepared using chemical or conventional methods; (2) AgNWs have a high purity. This physical template method does not introduce other substances, including metal ions, organic substances, and oxides, except for impurities in the target material itself; (3) It can be seen from some of the intersections in the image that different NWs can be perfectly cross-linked at the intersection. All the above three points have favorable effects on the electrical conductivity of the nanonetworks. The diameter of the AgNWs obtained under the given experimental conditions is 946 nm (as shown in Figure S1a in Supplementary Materials). The sputtering time for this sample was 3 min.

**Figure 3:**
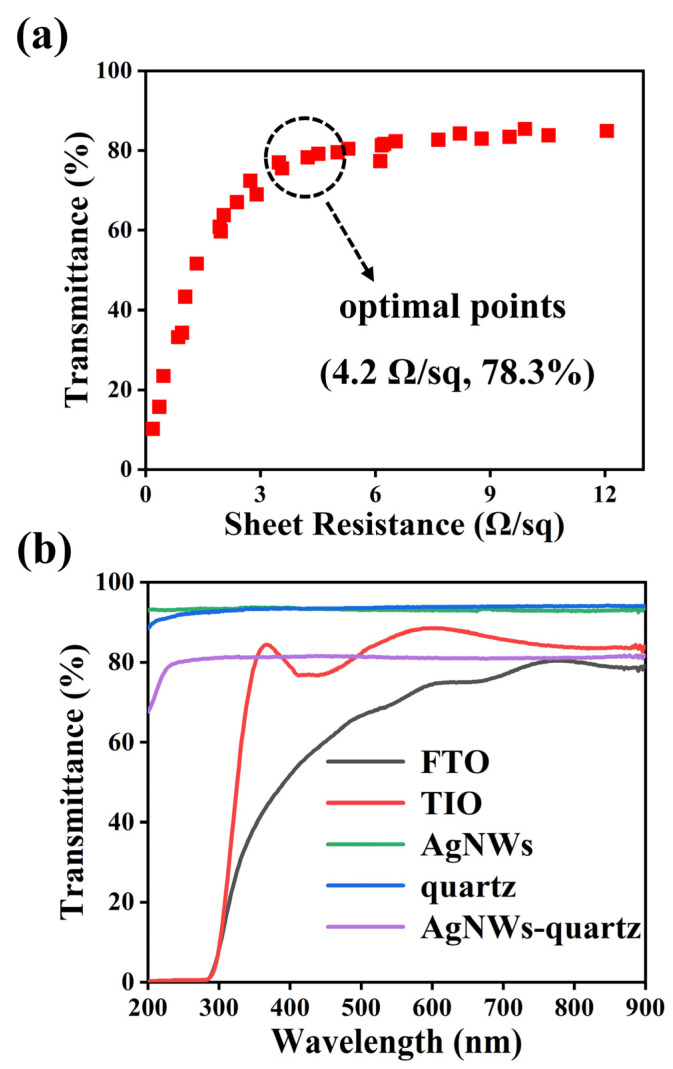
(**a**) Relation diagram of sheet resistance and transmittance of AgTCFs. (**b**) Transmittance curves of different TCFs.

The light transmission and electrical conductivity of the prepared AgTCF are affected by the diameter and density of AgNWs. We obtained a series of AgTCFs with different properties by varying the electrospinning time. As shown in Figure 3a, the sheet resistance of AgTCFs increased continuously from 10^−1^ Ω/sq to 10^1^ Ω/sq as the NW density decreased, and the light transmittance of the film rapidly saturated to about 80%. When applied to DSSCs and photoelectrochemical UV detectors, we can choose the data at the inflection point of the curve as the optimal electrode conditions. Figure 3b shows the transmittance curves of various TCFs in the UV–visible region. As is well-known, conductive oxide based TCFs such as FTO and ITO have strong absorption for UV light below 400 nm and cannot transmit light below 300 nm, which limits their application in UV devices. The transmittance in the visible region of FTO/ITO has weak fluctuations. The bare Ag nanonetworks we prepared have excellent transmission throughout 200–900 nm with a transmittance of about 94%. After transfer to the quartz substrate, the transmittance exhibits a slight decrease to about 80%. Compared with FTO/ITO, AgTCF has a more stable transmission across the entire wavelength band. It is important to note that the above data are just some of the typical results we obtained. The properties of AgTCF obtained using this method are influenced by the diameter, density, and length of the NWs, and the films can be optimized by changing the spinning substance, spinning time, spinning temperature, and sputtering parameters. In view of the advantages of AgNWs in three aspects, length, purity and connection method, we believe that the AgTCF obtained using this strategy can achieve the perfect transparent conductive properties.

Heat-resistance temperature is another key indicator of TCFs. The TCFs based on metal NWs have been widely studied; however, to the best of our knowledge, few of them have been successfully applied to photoelectrochemical devices. In addition to chemical instability and carrier recombination, the inability to withstand high temperatures is an important reason. Therefore, we investigated the heat resistance of AgTCFs by observing the changes in sheet resistance and microscopic morphology at different temperatures.

Due to the changes in surface energy, defect and face-to-volume ratio caused by the reduction in size, the heat resistance of AgNW will decrease with the decrease in diameter [14]. At the same time, the metal itself has impurities, twin boundaries, dislocation and other defects [15]; therefore, in general, the thermal behavior of AgNWs is not completely identical. The binding energy of the atoms on the surface of the NWs is smaller than that of the atoms on the interior, with fewer nearest neighbor atoms and weaker bonds; therefore, the melting of AgNWs starts from the surface [16]. When the temperature rises to a certain value, the NWs gradually melt and break. Eventually, to ensure the lowest surface energy, it starts to turn into spherical clusters.

**Figure 4:**
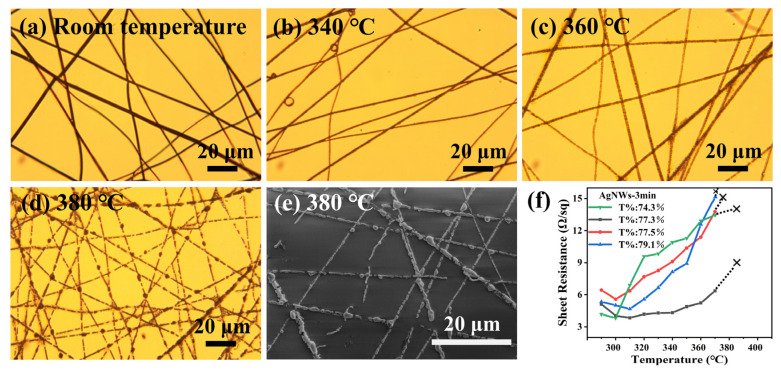
(**a**–**d**) Micrograph of AgNW films treated at different temperatures. (**e**) The FESEM morphology of AgNW films treated at 380 °C. (**f**) Relation diagram of temperature and sheet resistance of AgNWs. Where T% is the transmittance.

It can be seen from Figure 4a–d that the morphology of AgNWs starts to show slight changes when the heat treatment temperature reaches 360 °C. When the heat treatment temperature reached 380 °C, the AgNWs had completely fractured and agglomeration appeared. The SEM image shown in Figure 4e gives a clearer demonstration. The sheet resistance of AgNWs was measured with four probes. As shown in Figure 4f, the sheet resistance of the sample changes continuously with the increase in temperature and cannot be measured when the temperature exceeds 370 °C. The results showed that the heat-resistance temperature of AgNWs is about 370 °C. In order to obtain more accurate results, four samples with different transmittance were selected and measured, and a uniform conclusion, which is the same as above, was obtained.

### 3.2. The Ag/W and W/Ag Composite Nanonetwork TCFs

The heat-resistance temperature of the prepared AgTCF (370 °C) does not reach the phase transition temperature of common semiconductor materials used in photoelectrochemical devices; therefore, it needs to be further improved. Compositing with high melting point metal materials is an effective strategy.

**Figure 5:**
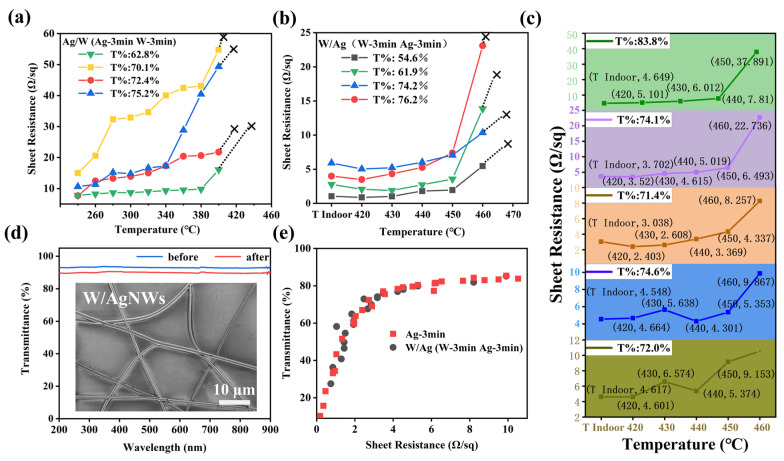
(**a**) Sheet resistance of Ag/WTCFs treated at different temperatures. (**b**) Sheet resistance of W/AgTCFs treated at different temperatures. (**c**) Test for heat resistance of W/AgTCFs at different W sputtering time (2, 3, 4, 5 and 6 min, respectively). Where T% is the transmittance. (**d**) Transmittance curves of AgNWs and W/AgNWs (without substrates). The inset is a SEM image of W/AgNWs. (**e**) Relation diagram of sheet resistance and transmittance of AgTCF and W/AgTCF.

Metal W is a typical high heat resistance metal; its melting point among all pure metals is extremely high, up to 3410 °C, but the electrical conductivity of WNWs is not outstanding. In this work, we successively sputtered Ag and W materials on PVP templates to form composite nanonetworks to optimize the heat resistance of the films. The samples obtained by sputtering Ag first and then W are referred to as Ag/WNWs (and accordingly, Ag/WTCFs). The samples obtained by sputtering W first and then Ag are referred to as W/AgNWs (and accordingly, W/AgTCFs). The light transmission, electrical conductivity and heat resistance of the composite TCFs were investigated (as shown in Figure 5). When the heat treatment temperature reaches a certain value, the melting state of metal layer changes, and the sheet resistance increases gradually with the increase in temperature until it cannot be measured with four probes.

High melting point metal coating low melting point metal, that is, W coating Ag (Ag/W) is the first composite structure we thought of. As shown in Figure 5a, Ag/WTCFs have the highest tolerance temperature of 400 °C. The heat-resistance temperature of the Ag/WTCFs obtained is 30 °C higher than that of the single-phase AgNW, indicating that the addition of metal W can improve the heat resistance. To our surprise, the W/AgTCFs exhibited better heat resistance, with an increase of 100 °C to 460 °C. It shows that the internal W plays an important role in improving the film. We therefore proceeded to vary the sputtering time of W (2, 3, 4, 5 and 6 min, respectively). The results are shown in Figure 5c. The change of W layer thickness did not significantly affect the heat resistance of the whole film, and only a slight decrease of 10 °C in the heat-resistance temperature was observed when the sputtering time of W was 2 min. Figure 5d shows that the transmittance of W/AgNWs is slightly lower than that of AgNWs due to the slight increase in diameter blocking more light (as shown in Figure S1b). The inset of Figure 5d shows that the morphology of W/AgNWs did not change significantly. We further tested the synergistic relationship between light transmission and electrical conductivity of W/AgTCFs (Figure 5e), and the results showed that it was not significantly different from the bare AgTCF; we believe that the above synergistic relationship depends mainly on the density of the electrospun template and the thickness of the Ag layer.

**Figure 6:**
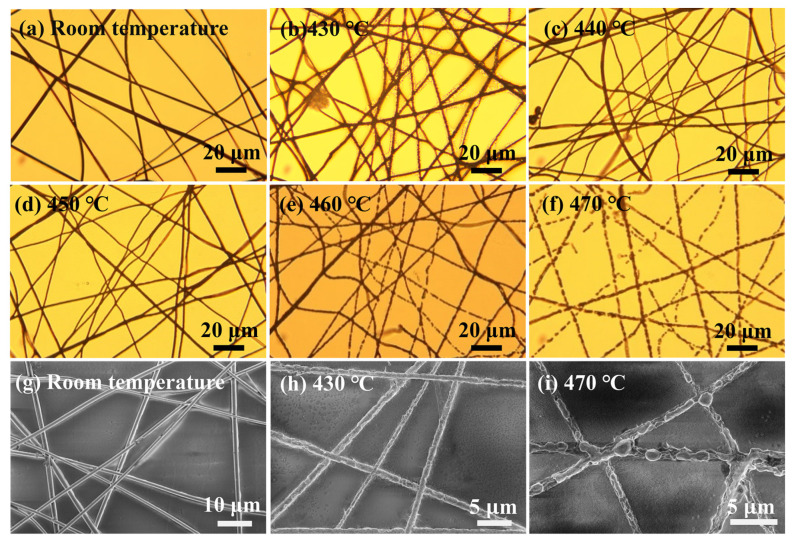
(**a**–**f**) The micrograph of melting state of W/AgNWs at different temperatures. (**g**–**i**) The SEM images of melting state of W/AgNWs at different temperatures.

As shown in Figure 6, we observed the morphological changes of W/AgNWs at different heat treatment temperatures using optical microscopy and SEM, and confirmed that the heat-resistance temperature of W/AgNWs is about 460 °C. Below 450 °C (including 450 °C), the NW still remains linear and continuous. When the temperature reached 460 °C, it was obvious that the morphology of some NWs began to melt and even appeared to break. When the heat treatment temperature reaches 470 °C, all of the NWs are fused.

### 3.3. Analysis of Thermal Stability

**Figure 7:**
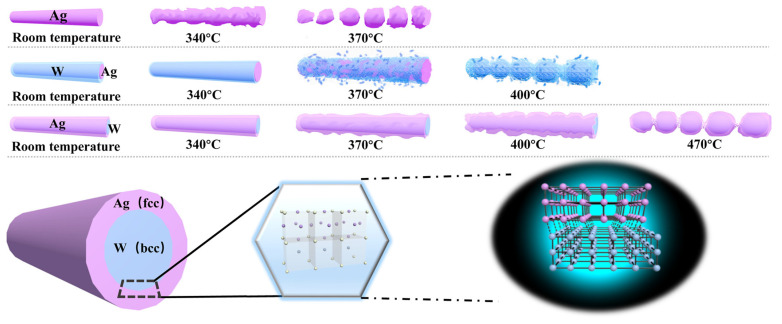
Schematic diagram of the melting processes and structures of different films.

Bare AgNWs, Ag/WNWs and W/AgNWs exhibit different thermal stability and melting processes. As shown in Figure 7, we have drawn a schematic diagram of the melting processes and structures of different films. The results obtained in the above thermal stability study are analyzed theoretically.

As shown in Figure 5a,b, the heat resistance of the composite TCFs was enhanced regardless of whether the added W metal was the outer or inner layer, which is called overheating phenomenon. In other words, the melt is restrained by some action during the non-uniform nucleation process. This is due to the three following reasons:

(1) A semi-coherent lattice relationship is formed at the interface of W and Ag, which changes the thermal vibration of atoms and inhibits the nucleation [17,18] and the growth of melted NWs. According to the mismatch degree of interface atoms, phase interfaces are divided into coherent, semi-coherent and non-coherent interfaces, which is defined as:

(1) $$\delta =\frac{a_{\alpha }-a_{\beta }}{a_{\alpha }}$$

where *δ* is the mismatch (constant), *a_α_* > *a_β_*, respectively, are the atomic spacing of crystals parallel to the interface on both sides. When *δ* < 0.05, the phase interface is coherent; when 0.05 < *δ* < 0.25, the phase interface is a semi-coherent interface; when *δ* > 0.25, the phase interface is an incoherent interface. The larger *δ* is, the greater the interfacial energy is. The crystal structure of Ag is face-centered cubic (fcc), and the crystal structure of metal W is body-centered cubic (bcc); then, heterogeneous interfaces can be formed. W and Ag are insoluble and cannot form compounds; therefore, sharp interfaces can be formed at atomic scale. According to the formula, the interface atomic mismatch of W and Ag is 0.23; therefore, the interface of the two phases belongs to the semi-coherent interface.

Solid phase transition is achieved through nucleation and growth. The difference of free energy between the old and new phases drives the formation of solid phase transition, while the interface energy and strain energy hinder the formation of solid phase transition [19,20]. According to the Lindemann criterion [21], melting is the vibrational lattice instability released when the root mean sheet displacement (rmsd) of atoms reaches a critical fraction of atomic spacing. When the crystal is in the thermal environment above the melting point, the melting occurs only when the amplitude of the thermal vibration reaches a critical fracture between atoms. The thermal vibration of the atoms on the semi-coherent interface is suppressed by the larger interfacial energy.

(2) When the liquid core is formed in the crystal, the change of the volume will cause the strain in the crystal, and the strain energy will increase, which hinders the occurrence of the solid phase transition [16,22,23,24]. This occurs mainly in Ag/WNWs.

(3) When melting occurs, the dynamic process at the interface is mainly atomic bond breaking [16]. For the composite nanonetworks, there are strong covalent bonds between the W atoms, which are the matrix. For coherent or semi-coherent interfaces, the lattice mismatch at the interface is small, and the chemical bonds between the atoms at the interface and the matrix have more or less ionic properties, which will lead to the fact that the thermal vibration at the interface is stronger than the inner W, and weaker than that of the outer Ag.

According to the above analysis, the heat-resistance temperature of Ag and W composite NWs should be between bare AgNW and WNW due to the effect of multiple interactions at the interface. The heat-resistance temperature of WNWs is 670 °C (Figure S2), so 400 °C and 460 °C are reasonable values.

When the magnetron sputtering conditions are the same (namely 150 W, 0.5 Pa, 3 min), the heat-resistance temperature of W/AgNWs (460 °C) is higher than that of Ag/WNWs (400 °C) for the following reasons.

The linear expansion coefficients of Ag and W are 19.2 × 10^−6^/K and 4.5 × 10^−6^/K, respectively. When the heat treatment is carried out from room temperature to high temperature, the expansion degree of the two phases is different, and the contraction degree is also very different after the temperature drops from high temperature. Compared with W phase, Ag has a larger shrinkage degree and a faster shrinkage speed. Therefore, when the temperature drops, great internal stress is generated at the interface. When the temperature cooling speed is too fast, the internal stress generated cannot be released [25]. At this time, Ag phase produces residual tensile stress and W phase produces residual compressive stress [26]. In the case of Ag/WNWs, the inner Ag layer gradually melts and expands under the action of high temperature, then breaks and finally changes into chain ball shape. In this process, the Ag layer expands when heated, and the expansion state is much higher than that of the W layer. This process leads to the premature destruction of the external W layer. In the case of W/AgNWs, when the temperature rises to a certain temperature, AgNWs splits into chain ball ahead of WNWs. The inside WNWs are gradually “pulled” broken by the surface tension of the molten Ag layer. Compared with Ag/WNWs, the overall heat resistance of W/AgNWs shows certain advantages without the dual effects of Ag thermal expansion and surface tension.

In addition, some interesting results should be noticed. (1) As shown in Figure 4f and Figure 5b, the sheet resistance of the films showed a certain degree of reduction at the beginning of the heat treatment, both for AgNWs and W/AgNWs. This is mainly because, firstly, the heat treatment improves the crystallinity of the AgNWs; secondly, the heat treatment makes the PVP melt and volatilize, and the AgNWs fuse at the crossover point. However, we did not observe a similar phenomenon in the Ag/WNWs shown in Figure 5a, which is due to the higher melting temperature of the outer W layer, which remains solid at the beginning of the heat treatment, separating the different AgNWs. This further demonstrates the advantage of W/AgNWs. (2) For Ag/WNWs, the sheet resistance of the film gradually increases when the heat treatment temperature reaches 260 °C, which is not found in AgNWs and W/AgNWs. As analyzed earlier, this is due to the fact that the coefficient of thermal expansion of Ag is much larger than that of W, and the stress generated in the inner layer after the temperature increase destroys the W layer prematurely. (3) In each set of heat treatment experiments, several samples with different characteristics were selected for testing, but the sample heat-resistance temperature did not change significantly, which further indicates that the interface is the most critical factor affecting the heat resistance of the composite system. (4) For W/AgNWs, increasing the thickness of the W layer can slowly increase the heat-resistance temperature of the whole film, but this is not possible in the experiment. The load-bearing capacity of the PVP NW network template is limited, and an excessively thick W layer will destroy the self-support of the template. (5) For all samples, increasing the thickness of Ag can effectively improve the electrical conductivity of the film and obtain a lower sheet resistance without bringing a significant impact on the light transmission. However, this would increase the roughness of the film and bring about an increase in cost. Therefore, we did not control this parameter in our experiments and uniformly selected a sputtering time of 3 min. However, we believe that there is still much room for improving the conductivity of AgNWs-based TCFs.

## 4. Conclusions

We prepared AgNW network-based TCFs using a special physical template method, and the films showed good transmittance in the UV–visible region. After optimization, their sheet resistance can be as low as below 4.2 Ω/sq. Benefiting from the characteristics of the preparation method, it is very convenient to compound AgNWs with other materials. Considering the demand of heat resistance for device applications, we prepared a variety of composite TCFs of Ag and W, and successfully increased the heat resistance of the films to 460 °C. Numerous phenomena in the study were analyzed theoretically. We believe that the key factors for the improved heat resistance are: (1) A semi-coherent lattice relationship is formed at the interface of W and Ag, which changes the thermal vibration of atoms and inhibits the nucleation and the growth of melted NWs. (2) The coefficient of thermal expansion of Ag is much larger than that of W. When the heat treatment is carried out from room temperature to high temperature, the expansion degree of the two phases is different. (3) The chemical bonds between the atoms at the interface have more or less ionic properties, which will lead to the fact that the thermal vibration at the interface is stronger than the inner W, and weaker than that of the outer Ag.

## Data Availability

Data is contained within the article or supplementary material.

## Supplementary Materials

The following supporting information can be downloaded at: https://www.mdpi.com/article/10.3390/nano13040708/s1. Figure S1: diameter statistics of Ag and W/Ag composite NWs; Figure S2: the melting state of WNWs at different temperatures.
