# Supplementary material for: High Temperature-Resistant Transparent Conductive Films for Photoelectrochemical Devices Based on W/Ag Composite Nanonetworks
Source: Nanomaterials (Basel). 2023 Feb 12;13(4):708. doi: 10.3390/nano13040708 (PMC9960394; doi:10.3390/nano13040708)
Supplement: Supplementary file 1 [file nanomaterials-13-00708-s001.zip › nanomaterials-2198142-supplementary.pdf]

High Temperature Resistant Transparent Conductive Films for Photoelectrochemical Devices Based on W/Ag Composite Nanonetworks

Menghan Liu 1, Peiling Ren 1, Hu Qiao 2, Miaomiao Zhang 1, Wenxuan Wu 1, Baoping Li 1, Hongjun Wang 1, Daobin Luo 1, Jianke Liu 1, Youqing Wang 1,*

[1] Research Center for Semiconductor Materials and Devices, Shaanxi University of Science and Technology, Xi'an 710021, China
[2] School of Mechatronic Engineering, Xi'an Technological University, Xi'an 710021, China

* Corresponding author. E-mail address: wangyouqing@sust.edu.cn

Supplementary Data

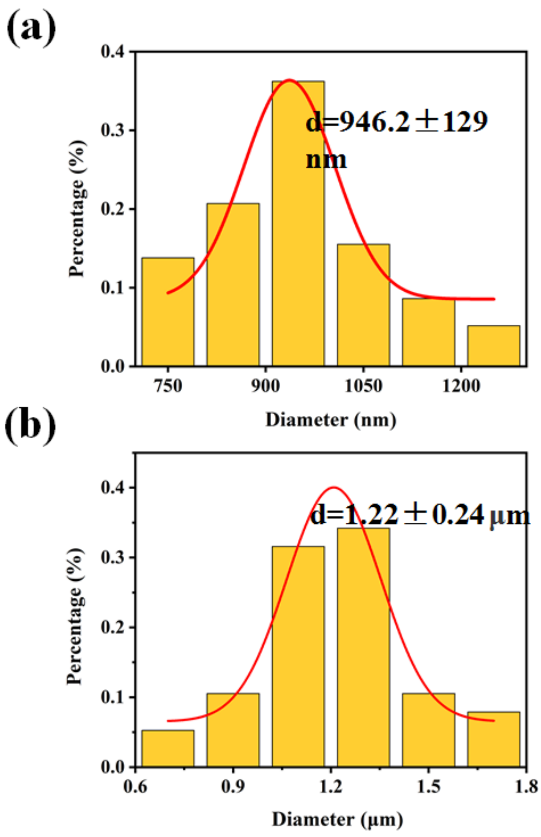

Figure S1. Diameter statistics of (a) Ag nanowires and (b) W/Ag composite

nanowires.

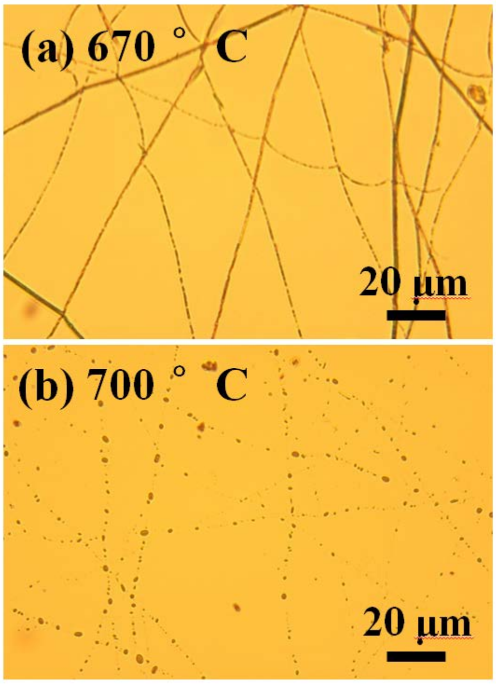

Figure S2. The melting state of W nanowires at different temperatures.
